# Supplementary material for: Kinesin-6 Klp9 plays motor-dependent and -independent roles in collaboration with Kinesin-5 Cut7 and the microtubule crosslinker Ase1 in fission yeast
Source: Sci Rep. 2019 May 14;9:7336. doi: 10.1038/s41598-019-43774-7 (PMC6517423; doi:10.1038/s41598-019-43774-7)
Supplement: Supplementary file 1 — Supplementary Information [file 41598_2019_43774_MOESM1_ESM.pdf]

Supplementary Information

Kinesin-6 Klp9 plays motor-dependent and -independent roles in collaboration with Kinesin-5 Cut7 and the microtubule crosslinker Ase1 in fission yeast

Masashi Yukawa and Masaki Okazaki, Yasuhiro Teratani, Ken'ya Furuta and Takashi Toda

Supplementary Table S1: Fission yeast strains used in this study

Supplementary Figure S1: Purification of the Klp9 protein for TIRF microscopy

Supplementary Figure S2: Genetic interactions between various *klp9* mutants and deletions of other MAP

Supplementary Figure S3: Spindle elongation rate during the period prior to anaphase B in the *cut7-122* mutant

Supplementary Table S1: Fission yeast strains used in this study

Strains	Genotypes	Figures used	Derivations
MO282	h^{-} *klp9-YFP-natR pcp1-CFP-hphR aur1R-Pnda3-mCherry-atb2 leu1 ura4*	2A-B	This study
MO330	h^{-} *klp9*^{rigor}*-YFP-natR pcp1-CFP-hphR aur1R-Pnda3-mCherry-atb2 leu1 ura4*	2A-B	This study
MO295	h^{-} *klp9-2-YFP-natR pcp1-CFP-hphR aur1R-Pnda3-mCherry-atb2 leu1 ura4*	2A-B	This study
MO100	h^{-} *cut12-GFP-ura4*$^{+}$ *aur1R-Pnda3-mCherry-atb2 leu1 ura4*	2C-E, 6D-F, S3A-B	This study
MO404	h^{-} *klp9::hphR cut12-GFP-ura4*$^{+}$ *aur1R-Pnda3-mCherry-atb2 leu1 ura4*	2C-E, 6D-F, S3A-B	This study
MO389	h^{-} *klp9*^{rigor}*-kanR cut12-GFP-ura4*$^{+}$ *aur1R-Pnda3-mCherry-atb2 leu1 ura4*	2C-E	This study
MO388	h^{-} *klp9-2-kanR cut12-GFP-ura4*$^{+}$ *aur1R-Pnda3-mCherry-atb2 leu1 ura4*	2C-E	This study
MY984	h^{-} *klp9::hphR leu1 ura4*	3A, 3C	This study
MO393	h^{+} *ase1::kanR leu1 ura4 his2*	3A, 3C, 4A, 5A, 5D, S2B-C	This study
MO286	h^{-} *klp9*^{rigor}*-kanR leu1 ura4*	3A-B	This study
MY1760	h^{+} *ase1::hphR leu1 ura4 his2*	3A-B, 4A, S2B-C	This study
513	h^{-} *leu1 ura4*	3B	Our lab stock
MY1321	h^{-} *klp9-2-kanR leu1 ura4*	3B	This study
MY1277	h^{-} *klp9*^{rigor}*-kanR ase1::hphR leu1 ura4*	3B-C	This study
MY1220	h^{-} *klp9-2-kanR ase1::hphR leu1 ura4*	3B-C	This study
MY990	h^{+} *cut7::bleR pkl1::natR leu1 ura4 his2*	3B-C, S2A, S2C	This study
MO164	h^{+} *klp9-2-kanR cut7::bleR pkl1::natR leu1 ura4 his2*	3B-C	This study
MY1462	h^{-} *klp9-GFP-kanR aur1R-Pnda3-mCherry-atb2 leu1 ura4*	4A-C, 5A, 5C	This study
MY1735	h^{-} *klp9*$^{\Delta 38C}$*-GFP-hphR aur1R-Pnda3-mCherry-atb2 leu1 ura4*	4A-C	This study
MO343	h^{-} *klp9*$^{\Delta 92C}$*-GFP-kanR aur1R-Pnda3-mCherry-atb2 leu1 ura4*	4A-C	This study
MO365	h^{-} *klp9*$^{\Delta 133C}$*-GFP-kanR aur1R-Pnda3-mCherry-atb2 leu1 ura4*	4A-C	This study
MO366	h^{-} *klp9*$^{\Delta 172C}$*-GFP-kanR aur1R-Pnda3-mCherry-atb2 leu1 ura4*	4A, 4C	This study
MY1727	h^{-} *klp9*$^{\Delta 234C}$*-GFP-kanR aur1R-Pnda3-mCherry-atb2 leu1 ura4 lys1?*	4A, 4C	This study
MO384	h^{-} *klp9*^{KARAKA}*-GFP-kanR aur1R-Pnda3-mCherry-atb2 leu1 ura4*	4A-C	This study
MO362	h^{-} *klp9*$^{\Delta Motor}$*-GFP-kanR aur1R-Pnda3-mCherry-atb2 leu1 ura4*	4A-C	This study
MY1331	h^{-} *klp9*$^{\Delta 38C}$*-GFP-hphR leu1 ura4*	4A, S2B-C	This study
MO338	h^{-} *klp9*$^{\Delta 92C}$*-GFP-kanR leu1 ura4*	4A, S2B-C	This study
MO319	h^{-} *klp9*$^{\Delta 133C}$*-GFP-kanR leu1 ura4*	4A, S2C	This study
MO337	h^{-} *klp9*$^{\Delta 172C}$*-GFP-kanR leu1 ura4*	4A, S2C	This study
MO316	h^{-} *klp9*$^{\Delta 234C}$*-GFP-kanR leu1 ura4*	4A, S2C	This study
MO369	h^{-} *klp9*^{KARAKA}*-GFP-kanR leu1 ura4*	4A, S2B-C	This study
MO368	h^{-} *klp9*$^{\Delta Motor}$*-GFP-kanR leu1 ura4*	4A, S2B-C	This study
MY1939	h^{+} *klp9-GFP-kanR cut12-GFP-ura4*$^{+}$ *aur1R-Pnda3-mCherry-atb2 leu1 ura4 his2*	4E, 5F	This study
MY1941	h^{+} *klp9*$^{\Delta 38C}$*-GFP-hphR cut12-GFP-ura4*$^{+}$ *aur1R-Pnda3-mCherry-atb2 leu1 ura4 his2*	4E, 5F	This study
MY1943	h^{+} *klp9*$^{\Delta 92C}$*-GFP-kanR cut12-GFP-ura4*$^{+}$ *aur1R-Pnda3-mCherry-atb2 leu1 ura4 his2*	4E, 5F	This study

MY1945	h^{+} klp9$^{\Delta 133C}$-GFP-kanR cut12-GFP-ura4^{+} aur1R-Pnda3-mCherry-atb2 leu1 ura4 his2	4E, 5F	This study
MY1947	h^{+} klp9$^{\Delta 172C}$-GFP-kanR cut12-GFP-ura4^{+} aur1R-Pnda3-mCherry-atb2 leu1 ura4 his2	4E, 5F	This study
MY1949	h^{+} klp9$^{\Delta 234C}$-GFP-kanR cut12-GFP-ura4^{+} aur1R-Pnda3-mCherry-atb2 leu1 ura4 his2	4E, 5F	This study
MY1951	h^{+} klp9$^{\Delta Motor}$-GFP-kanR cut12-GFP-ura4^{+} aur1R-Pnda3-mCherry-atb2 leu1 ura4 his2	4E, 5F	This study
MY1953	h^{+} klp9^{KARAKA}-GFP-kanR cut12-GFP-ura4^{+} aur1R-Pnda3-mCherry-atb2 leu1 ura4 his2	4E, 5F	This study
MO416	h^{+} klp9$^{\Delta CC1}$-GFP-hphR aur1R-Pnda3-mCherry-atb2 leu1 ura4 his2	5A-C	This study
MO411	h^{-} klp9$^{\Delta CC2}$-GFP-hphR aur1R-Pnda3-mCherry-atb2 leu1 ura4	5A-C	This study
MO405	h^{-} klp9$^{\Delta CC1}$-GFP-hphR leu1 ura4	5A, 5D, S2C	This study
MO410	h^{-} klp9$^{\Delta CC2}$-GFP-hphR leu1 ura4	5A, 5D, S2C	This study
MY1955	h^{+} klp9$^{\Delta CC1}$-GFP-hphR cut12-GFP-ura4^{+} aur1R-Pnda3-mCherry-atb2 leu1 ura4 his2	5E-F	This study
MY1957	h^{+} klp9$^{\Delta CC2}$-GFP-hphR cut12-GFP-ura4^{+} aur1R-Pnda3-mCherry-atb2 leu1 ura4 his2	5E-F	This study
YT317	h^{-} cut7-GFP-kanR sid4-mRFP-natR aur1R-Pnda3-mCherry-atb2 leu1 ura4	6A, 6C	This study
YT080	h^{+} cut7-122-GFP-kanR sid4-mRFP-natR aur1R-Pnda3-mCherry-atb2 leu1 ura4 his2	6A, 6D-F, S3A-B	This study
YT252	h^{-} cut7-GFP-kanR klp9::hphR sid4-mRFP-natR aur1R-Pnda3-mCherry-atb2 leu1 ura4	6A	This study
YT074	h^{+} cut7-122-GFP-kanR klp9::hphR sid4-mRFP-natR aur1R-Pnda3-mCherry-atb2 leu1 ura4 his2	6A, 6C-G, S3A-B	This study
YT344	h^{-} cut7-GFP-kanR klp9^{rigor}-GFP-hphR aur1R-Pnda3-mCherry-atb2 leu1 ura4	6A	This study
YT352	h^{-} cut7-122-GFP-kanR klp9^{rigor}-GFP-hphR aur1R-Pnda3-mCherry-atb2 leu1 ura4	6A, 6G	This study
YT287	h^{+} cut7-GFP-kanR ase1::hphR sid4-mRFP-natR aur1R-Pnda3-mCherry-atb2 leu1 ura4	6A	This study
YT299	h^{+} cut7-122-GFP-kanR ase1::hphR sid4-mRFP-natR aur1R-Pnda3-mCherry-atb2 leu1 ura4	6A, 6G	This study
MY1328	h^{-} klp9$^{\Delta 38C}$-GFP-hphR pkl1::natR leu1 ura4	S2A, S2C	This study
MO347	h^{-} klp9$^{\Delta 92C}$-GFP-kanR pkl1::natR leu1 ura4	S2A, S2C	This study
MO326	h^{-} klp9$^{\Delta 133C}$-GFP-kanR pkl1::natR leu1 ura4	S2C	This study
MO335	h^{-} klp9$^{\Delta 172C}$-GFP-kanR pkl1::natR leu1 ura4	S2C	This study
MO328	h^{-} klp9$^{\Delta 234C}$-GFP-kanR pkl1::natR leu1 ura4	S2C	This study
MY1762	h^{-} klp9^{KARAKA}-GFP-kanR pkl1::natR leu1 ura4	S2A, S2C	This study
MO359	h^{-} klp9$^{\Delta Motor}$-GFP-kanR pkl1::natR leu1 ura4	S2A, S2C	This study
MO417	h^{-} klp9$^{\Delta CC1}$-GFP-hphR pkl1::natR leu1 ura4	S2A, S2C	This study
MO421	h^{-} klp9$^{\Delta CC2}$-GFP-hphR pkl1::natR leu1 ura4	S2A, S2C	This study

*Strains were developed for this study unless otherwise specified.
his2=his2-245; *leu1=leu1-32*; *ura4=ura4-D18*.

Supplementary Figure S1. Yukawa *et al*.

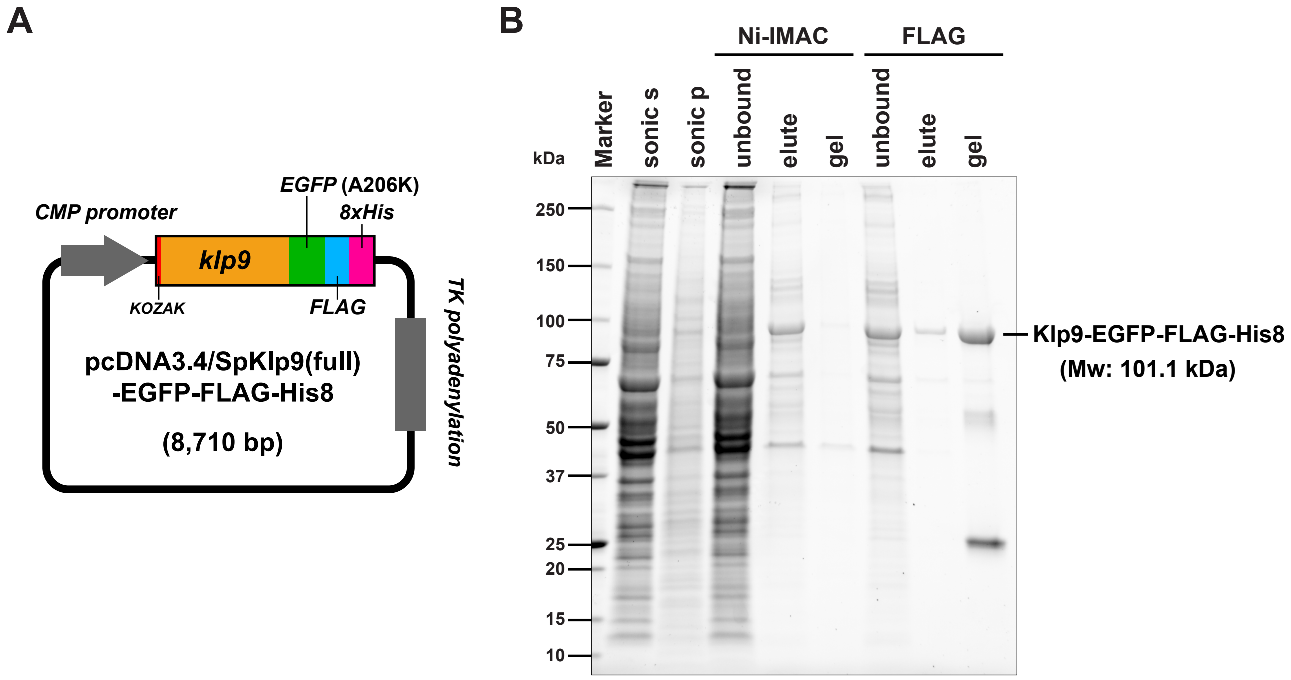

Supplementary Figure S1. Purification of the Klp9 protein for TIRF microscopy

(A) Diagram of a plasmid for transient expression full-length Klp9 in human cells.

(B) SDS-PAGE analysis (4-15%) of the full-length Klp9 protein. The protein bands were visualised by Stain-Free technology (Bio-rad). The right-most lane represents Klp9 preparations used for *in vitro* assays with TIRF.

Figure S2. Yukawa *et al.*

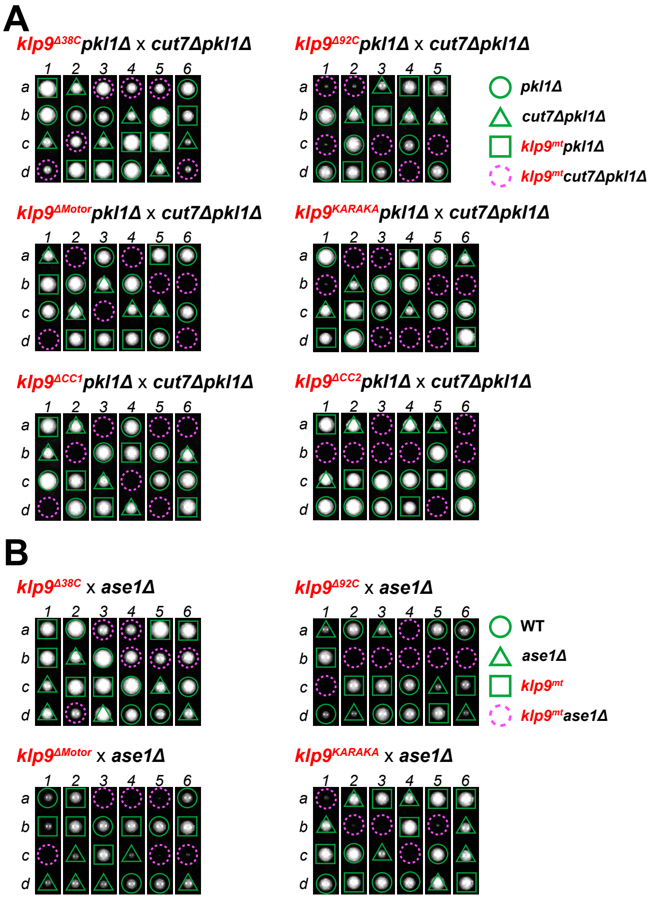

C

	Synthetic lethality	
***klp9* allele**	***cut7Δpkl1Δ***	***ase1Δ***
klp9+	V	V
Δ38C	V	V
Δ92C	SL	SL
Δ133C	SL	SL
Δ172C	SL	SL
Δ234C	SL	SL
ΔMotor	SL	SL
KARAKA	SL	SL
ΔCC1	SL	SL
ΔCC2	SL	SL

Figure S2. Genetic interactions between various *klp9* mutants and deletions of other MAP

(A, B) Tetrad analysis. Spores were dissected upon crosses between indicated strains Individual spores (a–d) in each ascus (1–8) were dissected on YE5S plates and incubated for 3 d at 27°C. Representative tetrad patterns are shown. **(A)** Circles, triangles and squares with green lines indicate *pkl1*Δ, *cut7*Δ*pkl1*Δ and *klp9^{mt}pkl1*Δ, respectively. **(B)** Circles, triangles and squares with green lines indicate wild type, *ase1*Δ, *klp9^{mt}*, respectively. Assuming 2:2 segregation of individual markers allows the identification of *klp9^{mt}cut7*Δ*pkl1*Δ **(A)** *klp9*Δ*ase1*Δ mutants **(B)** (indicated by dashed magenta circles). **(C)** A summary table indicating genetic interactions between various *klp9* mutants and *cut7*Δ*pkl1*Δ or *ase1*Δ. V stands for viable, while SL stands for synthetically lethal.

Figure S3. Yukawa *et al.*

A

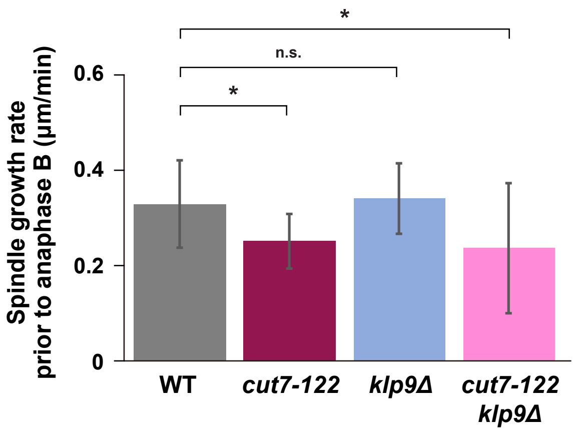

B

	Spindle growth rate (μm/min)			
	Phase I		Anaphase B	
WT	0.33 ± 0.09	(20)	0.75 ± 0.09	(20)
cut7-122	0.25 ± 0.06 *	(10)	0.67 ± 0.11 *	(16)
klp9Δ	0.34 ± 0.07 n.s.	(12)	0.38 ± 0.09 ****	(14)
cut7-122klp9Δ	0.24 ± 0.14 *	(13)	0.20 ± 0.06 ****	(20)

means ± SD P-values (n)

Figure S3. Spindle elongation rate during the period prior to anaphase B in the *cut7-122* mutant
(A) Spindle growth rates prior to anaphase B (phase I) in wild type (grey), *cut7-22* (dark magenta), *klp9Δ* (light blue), *cut7-122klp9Δ* cells (light magenta). **(B)** The velocity of spindle elongation during pre-anaphase B and anaphase B. The data are the same as in **(A)** and Fig. 6E. Data are given as means ± SD; *, P < 0.05; ****, P < 0.0001; n.s., not significant (two-tailed unpaired Student's t-test); Numbers in parentheses show the number of cells analyzed.
